# Angiotensin-(1-7) Provides Potent Long-Term Neurorepair/Neuroregeneration in a Rodent White Matter Stroke Model: Nonarteritic Ischemic Optic Neuropathy (rNAION)

**DOI:** 10.3390/cells14040289

**Published:** 2025-02-15

**Authors:** Kwang Min Woo, Yan Guo, Zara Mehrabian, Thomas Walther, Neil R. Miller, Steven L. Bernstein

**Affiliations:** 1 Weill Cornell Medical School, New York, NY 10021, USA; 2 Department of Ophthalmology and Visual Sciences, University of Maryland School of Medicine, Baltimore, MD 21201, USA; yanguo@som.umaryland.edu (Y.G.); zmehrabyan@som.umaryland.edu (Z.M.); sbernstein@som.umaryland.edu (S.L.B.); 3 Constant Therapeutics, LLC, Boston, MA 02210, USA; thomas.walther@medicalschool-berlin.de; 4 Medical School Berlin, Berlin 14197, Germany; 5 Department of Ophthalmology, Johns Hopkins University School of Medicine, Baltimore, MD 21205, USA; nrmiller@jhmi.edu

**Keywords:** stroke, white matter, nonarteritic anterior ischemic optic neuropathy (NAION), visual function, optic nerve, TXA127, Ang-(1-7), neurorepair, neuroregeneration

## Abstract

Nonarteritic anterior ischemic optic neuropathy (NAION) is an ischemic lesion of the anterior optic nerve (ON), currently untreatable due to the length of time from symptom onset until treatment. We evaluated angiotensin-(1-7) (Ang-(1-7)): the MAS1-receptor ligand, as a possible NAION treatment using the rodent NAION model (rNAION). Long-Evans rats were unilaterally rNAION-induced. One-day post-induction, lesion severity was quantified via optic nerve head (ONH) edema using spectral domain optical coherence tomography. Animals meeting rNAION induction criteria were randomized into (1) Subcutaneous Ang-(1-7) infusion for 28 days and (2) Vehicle. Visual function was assessed using both visual acuity and flash visual evoked potentials (fVEP). Tissues were collected >30d and RGC neurons were quantified by stereology. ONs were histologically examined for inflammation. Ang-(1-7) improved post-rNAION visual function. Ang-(1-7)-treated animals showed improved visual acuity (ANCOVA: *p* = 0.0084) and improved fVEP amplitudes (ANCOVA: *p* = 0.0378) vs vehicle controls. The relative degree of improvement correlated with ONH edema severity. Treated animals showed trends towards increased RGC survival, and reduced optic nerve inflammatory cell infiltration. Ang-(1-7) is the first agent effective ≥1 day after rNAION induction. Ang-(1-7) type agonists may be useful in improving long-term function and neuronal survival in clinical NAION and other forms of white matter ischemia.

## 1. Introduction

Nonarteritic anterior ischemic optic neuropathy (NAION) is a stroke of the anterior portion of the optic nerve, and the most common cause of sudden optic nerve-related vision loss [1]. NAION affects over 10,000 individuals per year in the US alone, making it a common cause of serious visual handicaps. While NAION can arise from multiple causes [1], it most commonly occurs in individuals with small optic nerve openings (‘disk at risk’) [2]. In this instance, vascular dysregulation is believed to cause capillary leakage and edema in the tightly restricted, inexpansive space at the optic nerve head (ONH) [2]. This edema results in a compartment syndrome with loss of circulation to the retinal ganglion cell (RGC) axonal bundles that emerge from the retina to form the optic nerve (ON). The subsequent axonal ischemia causes loss of communication with the higher central nervous system (CNS), RGC cellular stress, optic nerve inflammation and ultimately, loss of both affected RGC axons and their cell bodies. Vision loss from NAION ranges from regional visual field cuts to the loss of the entire visual field of the affected eye [3]. Approximately 15-20% of individuals who develop NAION in one eye develop NAION in the contralateral eye within five years [4]. There is currently no effective treatment.

We previously reported that a neuroregenerative treatment approach using a NOGO-66 targeting antibody (11C7Mab) generated a modest improvement in overall electrophysiological response [5]. However, the numbers of animals assessed were small (*n* = 6/group) and used flash visual evoked potentials (fVEPs) as the only test of visual function. Previous studies have shown that fVEP results using scalp electrodes are not as consistent and reproducible as results using transcranial electrodes and that using bilateral transcranial electrodes allows a more accurate comparison of visual function compared with scalp electrodes [6,7]. In addition, it seems clear that multiple methods of visual function analysis, rather than just fVEPs, should be incorporated into the evaluation of the results of late treatment strategies.

TXA127 is a pharmaceutical formulation of the angiotensinogen (aa 1-7) peptide fragment, Ang-(1-7). Ang-(1-7) is generated by angiotensin-converting enzyme-2 (ACE2) enzymatic digestion of angiotensinogen, which activates both angiotensin II receptors, as well as the MAS [8], and the MRGD receptors ([9]; for review see [10]). Ang-(1-7) counteracts the effects of Ang II in the renin–angiotensin system and its systemic effects include vasodilation, anti-thrombosis, and inhibition of cell growth. Ang-(1-7) also has been shown to be neuroprotective in a number of CNS disease models [11,12], including ischemia [13]. Ang-(1-7) exerts a neuroprotective effect even when administered 90 min orally after experimental middle cerebral artery occlusion (MCAO) [14]. Ang-(1-7) is hypothesized to work in the CNS by a number of mechanisms, including early suppression of pro-inflammatory cytokines [15], enhancement of neuroprotective macrophage development [16], and inhibition of pro-inflammatory microglia [17]. The regenerative effects of Ang-(1-7) also can be identified even when it is administered 2 months after the original ischemic insult [18]. Given that the optic nerve is part of the CNS and that both AngII and MAS1 receptors are widely expressed in the neuroretina [19,20], we hypothesized that Ang-(1-7) may exhibit therapeutic effects in experimental NAION even when its administration is delayed from the initial ischemic insult. In the current report, we evaluated the effect of subcutaneous Ang-(1-7) administration on both visual function and RGC survival of the adult rat when it is administered 1-day post rNAION induction.

## 2. Materials and Methods

We followed the ARRIVE 2.0 guidelines/Essential 10 for animal research.

### 2.1. Animals

All animal protocol experiments were approved by the institutional animal care and use committee (IACUC) and followed the recommendations of the Declaration of Helsinki. Pigmented Long-Evans (LE) outbred rats (250–275 g) were used, as albino strains such as Sprague-Dawley and Wistar have both diminished visual acuity compared with normally pigmented strains [21] and potential optic chiasm decussation anomalies. Animals used for osmotic pump implantation and cranial surgery were treated with both subcutaneous and sustained-release buprenorphine (Ethiqa XR; Fidelis Animal Health, North Brunswick Township, NJ, USA).

### 2.2. rNAION Induction

The induction procedure was previously described [22]. Briefly, animals were anesthetized with ketamine-xylazine and the pupils were dilated with 1% tropicamide and topically anesthetized with 0.5% proparacaine. A custom-made contact lens (Micro-R; Cantor & Nissel, Northamptonshire, UK) was placed on the eye. Animals were injected intravenously with rose Bengal dye dissolved in normal saline (2.5 mM; 1 mL/kg). Thirty seconds later, the intraocular portion of the optic nerve was illuminated with a 532nm laser light (Oculite GL; Iridex, Mountain View, CA, USA) at 50 mW power, 500 µm spot size, for 11 s. Power was checked at the eye by a laser power meter with a pyroelectric sensor (Coherent, Saxonburg, PA, USA).

### 2.3. Optic Nerve Head Imaging-Optic Nerve Edema Analysis

One-day post-rNAION induction, animals were re-anesthetized with ketamine-xylazine, their pupils dilated, and their corneas topically anesthetized. Spectral domain-optical coherence tomography (OCT; Heidelberg-Spectralis; Heidelberg, Germany) with a 28-diopter correcting rodent lens was used to image the intraocular optic nerve and to assess the severity of ONH edema. Mean ONH edema was based on the mean cross-sectional diameter of three contiguous central scans, as previously described [23]. The same Micro-R custom-made contact lens was used to generate the cross-sectional scans. Results were compared with the mean maximum diameters of the contralateral (uninduced eye) obtained from each animal. Animals with mean ONH edema < 450 µm were eliminated from the study prior to randomization and treatment based on previous studies by Guo et al. that showed 2-day ONH edema > 500 µm consistently resulted in RGC loss whereas less edema resulted in extremely variable RGC loss [23].

### 2.4. Ang-(1-7) and Vehicle Treatments

Anesthetized threshold animals with ≥450 µm mean ONH diameter 1d post-induction were immediately used for osmotic pump implantation. Animals were paired for equivalent edema levels, and randomized: one animal from each pair was used for either Ang-(1-7) or vehicle implant. Post-imaging, anesthetized animals were administered subcutaneously an initial loading dose of either Ang-(1-7) (1 mg/kg SC) or an equivalent volume of vehicle (sterile pH 7.4 PBS), then prepped aseptically for surgery using a 10% povidone scrub followed by a 10% povidone solution, alcohol rinsed, and dried. A midline incision between the shoulder blades was made, and an Alzet pump 2ML4 (Durect Corp, Cupertino, CA, USA) was inserted subcutaneously with additional local anesthesia (1% lidocaine). 2ML4 pumps deliver 2.5 μL/hr for 28 days, to yield 350 µg/day per animal. Animal IDs were then masked to the individual responsible for electrode implantation and testing (K.W.).

### 2.5. OptoMotry-Based Visual Acuity Assessment

Beginning 22 days post-induction, we measured visual acuity (VA) in unsedated animals using a virtual optokinetic system (OptoMotry: CerebralMechanics, TO, Canada), using a rat pedestal [24]. Rats were placed on a stable elevated platform in the center of a square surrounded by four monitors that projected a continuously moving sine-wave black-and-white grating. We assessed the ability of a rat to resolve a given spatial frequency by identifying when the animal turns its head at a constant speed in either a clockwise (left eye) or counterclockwise (right eye) direction. The VA of both eyes of each animal were evaluated by three independent trials, on separate days, with each eye’s estimated maximum VA elicited as a single measure using the staircase parameter, with a starting step size of 0.050 cycles/degree, based on the maximum reversal number (8) from any individual trial, and a column speed of 20. After each reversal, the step size was decreased by half. Three daily trials were performed on each animal, beginning on day 21 post-induction, and the result with the greatest level of acuity was considered maximal acuity.

### 2.6. Transcranial Electrode Implantation

As described by Woo et al. [7], bilateral screw electrodes were implanted over the occipital prominence a day post-OptoMotry, which occurred around 25 days after rNAION induction. Animals were anesthetized using ketamine/xylazine, then topically anesthetized subcutaneously using 1% lidocaine, with buprenorphine analgesia. The animal then was placed into a stereotactic instrument on a heating pad. The skull skin was incised at the midline and retracted, following which bilateral skull burr holes were made at stereotactic locations of the V1 visual cortex using published parameters [25], using a motorized surgical drill with care not to penetrate the dura. Two stainless steel pan-head screws (5/16″ length; catalog #11197; Minitaps, Seattle, WA, USA) were embedded, one over each visual cortex, and fixed in place with dental cement. The skin then was closed using stainless steel sutures. Following long-term analgesia administration (Ethiqua-XR), animals were placed on a warming pad until recovery from anesthesia and allowed to recover from surgery for at least 3 days prior to testing. A total of 4 animals (1/26 in the vehicle group and 3/24 in the treatment group) were lost during surgical electrode implantation at 25 days post-induction).

### 2.7. Visual Evoked Potentials

As described by [7], animals were dark-adapted overnight and then re-anesthetized using inhaled isoflurane followed by ketamine-xylazine in dim red light. Pupils were dilated using 1% cyclopentolate and 2.5% neosynephrine. Animals were placed on the prewarmed Celeris electrophysiology testing platform (Diagnosys LLC, Lowell, MA, USA). Combined corneal electrodes and light-emitting diode stimulators were placed on the eyes using Systane gel drops (Alcon, Ft Worth, TX, USA). Alligator microclips were hooked onto the two skull electrodes. Fifty simultaneous electroretinography (ERG) and fVEP flashes from each eye were averaged, and the visual function of the rNAION-induced eye compared with the contralateral healthy eye was calculated from fVEP results using a formula described by [7]. fVEP measurements were repeated twice for each eye within the same session. After the procedure, the eyes of each animal were covered with ophthalmic triple antibiotic ointment with dexamethasone, and the animal was placed in a warm cage and allowed to recover from anesthesia. ERG results were used to identify and exclude rats with possible retinal ischemia. Rats were excluded if the maximum ERG b-wave amplitude of the rNAION-induced eye was less than 50% of that of the contralateral non-induced eye.

### 2.8. Tissue Isolation

Following all testing, animals were euthanized using CO_2_ inhalation and then decapitated. Eyes and optic nerves were rapidly isolated, and the corneas were incised with a 26 ga needle and placed in 4% paraformaldehyde-phosphate buffered saline pH 7.4 (PFA-PBS). Tissues were post-fixed overnight, and the retina then was dissected and removed for whole-mount immunohistology. The ONH along with 1.5 mm of the adjacent anterior optic nerve was dissected from the sclera, and a more distal portion of the optic nerve also was removed. Both were then cryopreserved in 30% sucrose-PBS, embedded in OCT and flash-frozen at −80 °C. ONH and optic nerves were cross-sectioned at 10 µm thickness and stored at −20 °C until use.

### 2.9. Immunohistochemistry

Whole retinae were immunoreacted using goat polyclonal anti-Brn3a (Pou4f1) (sc-31984; RRID AB_630987; Santa Cruz Chemicals, Santa Cruz, CA, USA) at 1:1000 dilution. Macrophages and microglia were detected using rabbit polyclonal anti-Iba1 (RRID:AB_839504; Wako Chemicals USA, Inc., Richmond, VA, USA) at 1:500 dilution. Newly generated macrophages were identified using mouse monoclonal anti-CD68/ED1 (RRID:AB_1141557; Abcam, Cambridge, MA, USA), at 1:100 dilution. Retinae were immunostained using Cy3-labeled donkey anti-goat polyclonal antibody 1:500 (Affinipure, Cat 705-005-003; RRID AB_2340384; Jackson Immunoresearch, West Grove, PA, USA). Optic nerve sections were evaluated using IBA1 (for inflammatory cells) and CD68 (for extrinsic macrophages. Following primary antibody binding and wash, sections were reacted with the appropriate fluorescent-labeled secondary antibodies (Jackson Immunoresearch). Confocal microscopy was performed using a Leica 300 confocal microscope, and standard fluorescent microscopy was performed using a Keyence BZX-710 computerized microscope.

### 2.10. RGC Stereology

RGC quantification was performed using randomized computer-driven stereology. Brn3a(+) immunohistochemical staining was performed on whole retinae [26], and RGCs were counted from flat-mounted retinae using a Nikon E300 fluorescent microscope with a computer-driven, motorized stage. The RGC ratio (RGCs/total retinal area) ratio was calculated for each eye, and the treatment difference was calculated for each induced animal.

### 2.11. Statistical Calculations

Differences between ONH edema, OptoMotry-based visual acuity, fVEP-based visual function, and RGC survival between the Ang-(1-7)- and vehicle-treated groups were compared with unpaired two-tailed student’s *t*-test. [27,28]. We used analysis of covariance (ANCOVA) to compare the three dependent variables-visual acuities, fVEP-based visual function, and RGC survival-with treatment status, Ang-(1-7) vs vehicle, as a categorical independent variable, and ONH edema as a covariate. ANCOVA was performed to assess any differences in regression lines in visual acuity or fVEP-based visual function (see Section 3.2 and Section 3.3). A Shapiro-Wilk test also was performed to determine whether residuals between the observed and modeled values were normally distributed [28,29] and Levene’s test was used to evaluate the homogeneity of variance between the two rNAION-induced groups [27,28] to meet the assumptions for ANCOVA. All outcomes that underwent ANCOVA analysis showed normal distribution and had homogeneity of variances across different groups. A Mann–Whitney U test was used to evaluate densitometic differences in inflammation between optic nerve histological sections with similar degrees of RGC loss. Python and GraphPad Prism (GraphPad Software; Boston, MA, USA) were used to perform statistical analysis. Values in the paper are presented as mean ± standard error of the mean (SEM) unless otherwise specified [28,29].

## 3. Results

### 3.1. Optic Nerve Edema Following rNAION and Randomization

**Figure 1:**
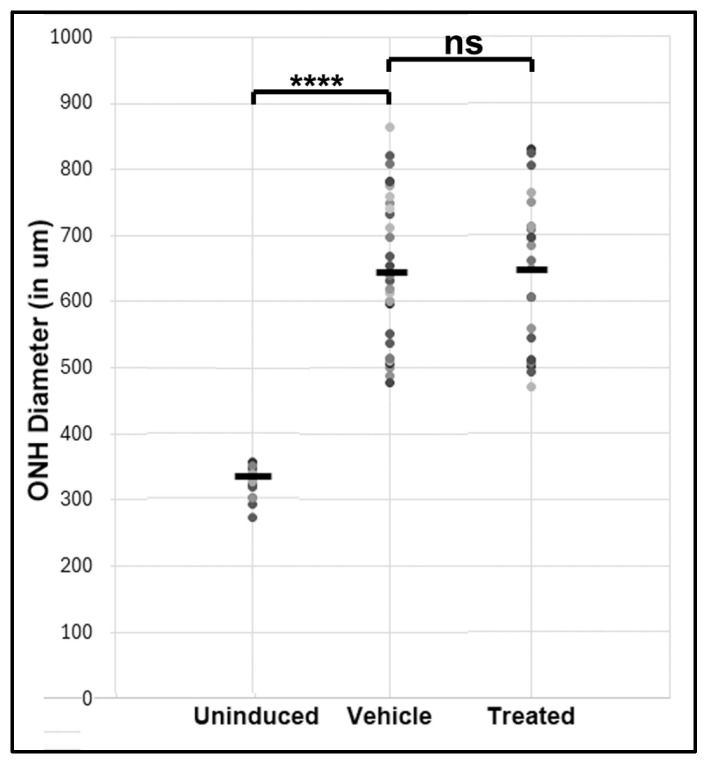
ONH edema results in 1d post-rNAION for both rat groups prior to treatment (both sexes). Mean ONH edema for each animal was based on results for the three largest contiguous diameters measured by SD-OCT and shown in microns (µM). Mean ONH diameter of 16 uninduced (contralateral eyes used for comparison). Animals were eliminated if ONH edema was ≤450 µm. Vehicle: *N* = 26; Treatment: *N* = 24. The relative spread of the edema is similar in both groups. There is a significant difference in ONH diameter between uninduced and induced eyes (**** *p* < 0.0001; two-tailed *t*-test). No significant (ns) difference was seen in the mean ONH diameter between the two randomized rNAION-induced (Vehicle and Treated) groups.

Mean ONH edema post-rNAION for randomized vehicle and treated groups are shown in Figure 1. Edema values were 660 ± 22.9 µm (*N* = 26) for vehicle and 649 ± 26 µm (*N* = 24) for treated animals, respectively (*p* = 0.735). The mean ONH diameter in the uninduced eye of all animals was 323 ± 5.8 µm (*N* = 16 animals).

### 3.2. Ang-(1-7) Improves Visual Acuity Following rNAION: OptoMotry

**Figure 2:**
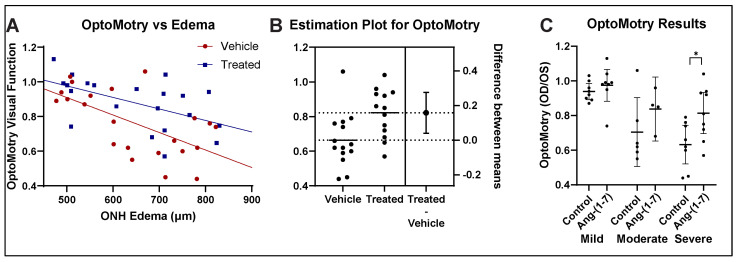
Ang-(1-7) improves visual acuity by OptoMotry after rNAION. (**A**) Regression analysis using analysis of covariance (ANCOVA). Regression lines for both vehicle- (*n* = 25) and Ang-(1-7)-treated (*n* = 21) are shown. ANCOVA reveals a significant difference in the intercepts between Ang-(1-7) and vehicle-treated rats. (F(1, 40) = 7.68, *p* = 0.0084) (**B**) Estimation plot for OptoMotry for animals with moderate-severe edema. The difference between treated and vehicle yielded an overall improvement of 16% for Ang-(1-7)-treated animals, compared with vehicle-treated animals with moderate-severe ONH edema, defined as edema ≥600 μm (t(25) = 2.753, *p* = 0.0108). (**C**) Subgroup analysis of Ang-(1-7) treatment effects post-rNAION. Ang-(1-7) provides an increasingly robust improvement in animals with progressively more severe ONH edema. There was a 3.6% improvement in the mild group (450–599 µm), a 13% improvement in the moderate group (600–699 µm), and an 18% improvement in the severe group (700–850 µm). (* *p* < 0.05).

We compared visual acuity in the rNAION-induced eye with the uninduced eye as a ratio of visual acuity measured in cycles per degree (c/d) in the rNAION-induced eye to the visual acuity in the control eye. Overall results for both vehicle- and Ang-(1-7)-treated animals are shown in Figure 2A [28]. Ang-(1-7)-treated animals performed significantly better in the rNAION-induced eye compared with the vehicle-treated animals with ONH edema as a covariate (ANCOVA, F(1, 40) = 7.68, *p* = 0.0084).

Ang-(1-7) treatment resulted in a mean 10% overall group improvement, compared with vehicle [unpaired *t*-test, t(44) = 1.994, *p* = 0.0523]. In animals with either moderate- or severe-ONH edema (≥600 µm), the increase in VA compared with vehicle-treated animals with similar amounts of edema was even more profound (Figure 2B), with an increase of ~0.20% (t(25) = 2.753, *p* = 0.0108). Thus, Ang-(1-7) treatment improves VA in rNAION when administered 1 day post-rNAION induction.

We further segregated rNAION-affected eyes into the relative severity of ONH edema (mild: 450–599 µm; moderate: 600–699 µm; severe: 700–850 µm) and compared the effects of Ang-(1-7) for each subgroup against the equivalent vehicle treatment subgroup (Figure 2C). Ang-(1-7)’s relative treatment efficacy improved the greater the severity of ONH edema, with 3.6% improvement in the mild group, 13% improvement in the moderate group, and 18% improvement in the severe group. Relative efficacy in improving VA was greatest for eyes with severe ONH edema.

### 3.3. Ang-(1-7) Improves Visual Function After rNAION: fVEP Amplitude Analysis

**Figure 3:**
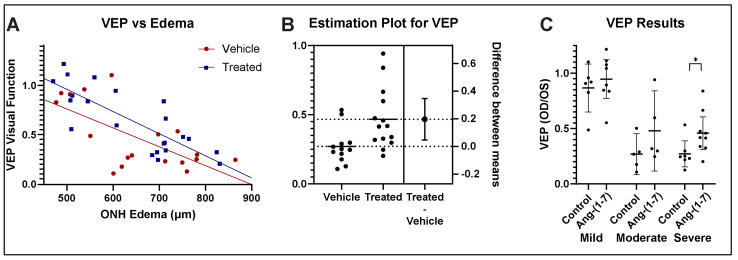
Ang-(1-7) administered 1 day after rNAION improves fVEP-based visual function as measured using waveform amplitudes: Analyses. (**A**) fVEP vs Edema regression analysis using ANCOVA. There is a significant difference in the intercepts between Ang-(1-7)- and vehicle-treated rats (F(1, 37) = 4.64, *p* = 0.0378). (**B**) Estimation plot for fVEP differences for a vehicle vs treated animals with moderate-severe ONH edema. There is a 20% overall improvement in the fVEP of treated animals with moderate-severe ONH edema, defined as edema ≥600 µm. (**C**) Stratified fVEP results for mild (450–599 µm), moderate (600–699 µm) and severe (700–850 µm) ONH edema. There was an 8.0% improvement in the mild group, a 21% improvement in the moderate group, and a 19% improvement in the severe group. Ang-(1-7) neuroregenerative/neuroreparative effects are strongest in animals with a moderate-severe amount of ONH edema. (* *p* < 0.05).

fVEP is widely used to evaluate changes in visual function following various conditions [30,31]. Ang-(1-7)-treated animals demonstrated improved visual function with respect to waveform amplitudes measured at the visual cortex, compared with vehicle-treated animals (Figure 3A) (ANCOVA, F(1, 37) = 4.64, *p* = 0.0378). Ang-(1-7) treatment, therefore, resulted not only in a significant improvement of visual acuity but also in a significant improvement of the fVEP in an overall significant improvement in visual function. The ANCOVA analysis of fVEP-based visual function revealed that Ang-(1-7)-treated animals demonstrated improved visual function with respect to waveform amplitudes measured at the visual cortex, compared with vehicle-treated animals (Figure 3A) (F(1, 37) = 4.64, *p* = 0.0378). Ang-(1-7) treatment, therefore, resulted not only in a significant improvement of visual acuity but also in a significant improvement of the fVEP in an overall significant improvement in visual function.

Ang-(1-7) treatment in animals with moderate-to-severe edema resulted in a 20% improvement in amplitudes compared with vehicle treatment (unpaired *t*-test, t(24) = 2.703, *p* = 0.0124). Overall changes in fVEP amplitude vs severity of ONH edema were stratified into comparative fVEP results from animals with mild, moderate, and severe ONH edema (Figure 3C), with an 8% improvement in the mild group, a 21% improvement in the moderate group, and a 19% improvement in the severe group. Ang-(1-7)’s effect was found to be greatest in eyes with the most severe disease.

### 3.4. Ang-(1-7) Effects Are Not Explained by Increasing RGC Survival

**Figure 4:**
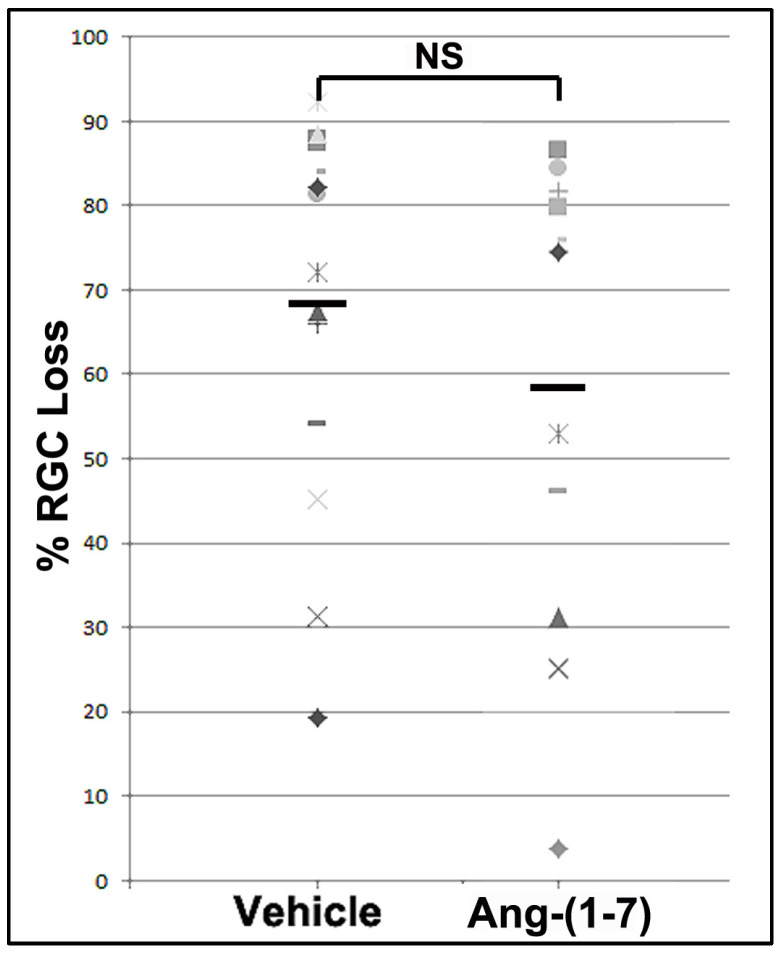
RGC loss in vehicle- and Ang-(1-7)-treated animals. RGCs were quantified by stereology. There is a similar distribution of RGC loss in both samples. The mean value for RGC loss in vehicle-treated animals was 74.29 ± 5.39% (*n* = 17), whereas Ang-(1-7)-treated animals yielded a mean RGC loss of 63.09 ± 7.17% (*n* = 14). This difference is not significant (NS: nonsignificant; unpaired *t*-test: *p* = 0.219).

Previous studies using RGC neuroprotectives have not been found to improve overall RGC survival when administered 1 day or later after the insult. Neuroregeneration is not based on neuroprotection and is not hypothesized to be directly responsible for overall functional change. Nevertheless, we performed RGC stereology on a randomized subset of eyes in both groups, with sufficient power for analysis. Figure 4 shows overall RGC comparisons. The mean RGC loss for vehicle-treated animals was 74.29 ± 5.39% (*n* = 17 randomly selected animals), whereas the mean RGC loss for Ang-(1-7)-treated animals was 63.09 ± 7.17% (*n* = 14 animals). This difference was not significant (unpaired *t*-test: (t(29) = 1.257, *p* = 0.219)

### 3.5. Ang-(1-7) Treatment Effects on Cellular ONH Inflammation After rNAION Induction

**Figure 5:**
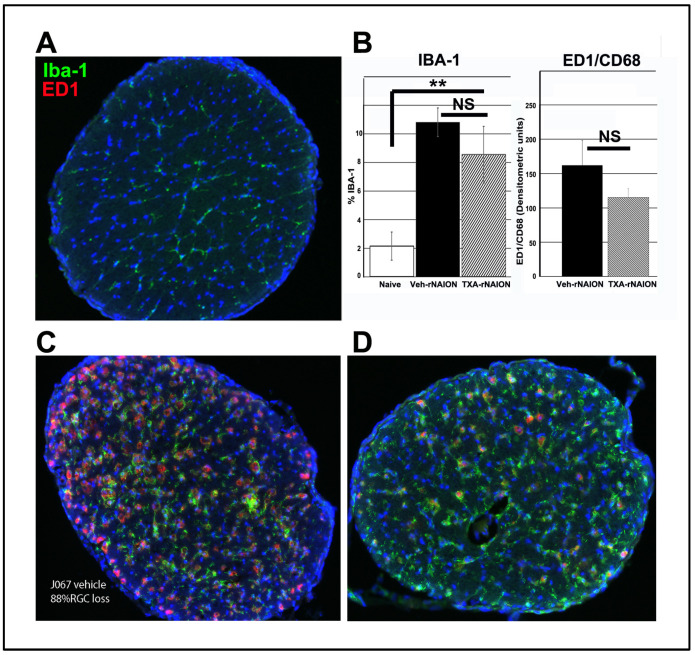
Ang-(1-7) effects on late cellular inflammation after rNAION. All tissues from animals 30d post-treatment. Only animals with similar levels of RGC loss were used for comparing vehicle- and Ang-(1-7)-treated responses. (**A**) Immunostaining of rat control ON (Green: Iba-1; Red: ED1/CD68). Ramified inactive microglia are distributed throughout the ON diameter, with little ED1 activity. (**B**) Quantification of inflammation: Comparison graphs. Vehicle-treated rNAION-induced ON has ~5 times the Iba-1 expression of naïve (10.7 ± 0.7% vs 2.2 ± 0.2% naïve). Iba-1 expression in Ang-(1-7)-treated animals is 8.5 ± 1.0% sem (*n* = 5/group). The difference between naïve and rNAION-induced animals treated with vehicle or Ang-(1-7) is highly significant (*p* = 0.003 for Ang-(1-7)-treated animals; the lower value). The difference between vehicle and Ang-(1-7) treatment is non-significant (Mann–Whitney two-tailed U test, *p* = 0.211). ED1/CD68 expression is shown in densitometric units; ED1 expression in naïve ONH is virtually null. The pattern of ED1/CD68 expression between vehicle and Ang-(1-7) treatment was similar to that seen with Iba-1 (N = 7/group; nonsignificant (NS), Mann–Whitney 2-tailed U test: *p* = 0.522). (** *p* < 0.01, ns = no significance) (**C**) Immunostaining of an rNAION-induced vehicle-treated animal. RGC loss at 30d = 88%. The majority of microglia/macrophages are hypertrophic/active, with strong upregulation of Iba-1 activity. ED1 activity is also elevated, confirming the activation of the majority of inflammatory cells. (**D**) Immunostaining of an rNAION-induced Ang-(1-7)-treated animal. RGC loss = 84%. The overall expression of both Iba-1 and ED1/CD68 is considerably lower than that seen in vehicle-treated rNAION-induced ON.

We previously determined that early cellular invasion (extrinsic macrophages) occurs within hours after CNS insult [32]. By 30 days post-induction, the majority of inflammatory cells in the ONH presumably represent microglia. We performed immunohistochemical analysis on a subgroup of vehicle- and Ang-(1-7)-treated animals matched with similar ONH edema values, 30 days post-induction, using ED1+ (activated macrophages and microglia) and IBA1(+) (macrophage/microglia specific) in more than 30 days post-rNAION induced ONHs (*n* = 5/group). ONHs from naïve animals reveal low levels of IBA1 signal approximately evenly distributed throughout the diameter of the tissue (Figure 5A), and there was no detectable CD68 (indicative of newly generated macrophages) signal in this group. Both rNAION-induced groups (Figure 5C: Vehicle-treated, and Figure 5D: Ang-(1-7)-treated) had increased levels of IBA1 and CD68/ED1 signal. ONHs from Ang-(1-7)-treated animals with equivalent ONH edema levels had slightly lower IBA1(+) and ED1(+) signal intensity than those of vehicle-treated animals (compare 5C with 5D). However, densitometric analysis revealed that any differences in IBA1 nor CD68 were statistically nonsignificant (Graph, Figure 5B; compare vehicle and Ang-(1-7) treatment groups). ONHs from Ang-(1-7)-treated animals had signal intensity of 8.54 ± 1.0% vs 10.8 ± 0.7% for vehicle-treated animals. This suggests that Ang-(1-7) treatment modestly suppresses post-ON infarct cellular inflammation, even when animals have similar amounts of initial lesion severity.

## 4. Discussion

Therapeutic approaches to NAION treatment fall roughly into two categories: neuroprotective and neuroregenerative. Neuroprotective approaches focus on early RGC preservation, whereas neuroregenerative approaches attempt to improve function independent of RGC loss. A number of neuroprotective agents have been shown to be effective in animal models of NAION when administered either before or shortly after NAION induction. [33,34,35] However, later (≥1d) treatment has shown no protective effects, (Bernstein, PGJ_2_ unpublished data) [35]. Similarly, human NAION clinical trials that have focused on RGC preservation (QRK201trial) have failed to show overall significant improvement, potentially because the time between NAION symptom onset and treatment initiation is too long to prevent RGC death. Successful NAION treatment strategies later in the disease course are thus likely to focus on neuroregenerative, post-ischemic functional enhancement.

In the rNAION-white matter stroke model, Ang-(1-7) (TXA127) represents the first compound that is functionally effective when administered at least 1 day after axon ischemic induction. Until now, NAION clinical treatment trials employing a double-blind treatment approach have not demonstrated a statistically successful outcome in improving visual function. NAION treatment trials incorporate individuals who typically present at least a day after onset. Similarly, previous reports using the rNAION model have failed to identify effective treatments when treatment is delayed more than 1-day post-induction [35]. This may be in part because the vast majority of previous approaches have focused on neuroprotective strategies that prevent RGC death. Activation of mechanisms associated with RGC death likely begins quite soon after axon ischemia, and an early ‘decision point’ within the neuron occurs, based on multiple pathways [36]. After this point, it becomes increasingly difficult to reverse a neuron’s degenerative course. Neuroregenerative/neuroreparative approaches that enhance residual function after the initial wave of RGC death thus represent an alternative treatment approach.

Previous neuroreparative/neuroregenerative approaches to the treatment of NAION have included inflammatory modulation [37], blockade of post-ischemic demyelination [5], alteration of the vascular supply of the affected ischemic penumbra [37], and enhanced responsiveness of surviving RGCs [38]. Additional, as yet unknown factors may be accessible to neuroregenerative/neuroreparative manipulation. Ang-(1-7) treatment has been previously shown to be effective in other CNS ischemic lesions [39] Its effects are known to include both immunomodulation and alteration in progenitor development [40,41].

In addition to Mas1, AngII and MRGPRD receptors have also been implicated in Ang1-7 function [10,42,43]. Previous studies have already demonstrated Mas1 expression in the eye [44]. Similar to many GPCRs, Mas1 has also been reported to transduce its signals by heterodimerization, e.g., with angiotensin II type 1 receptor (AT1R) and angiotensin II type 2 receptor (AT2R) [10]. We previously noted that although few RGCs express MAS, unstressed RGCs express low levels of AGTR2 in 29 subclasses, and ON-crush stressed RGCs strongly upregulate AGTR2 in 40 subclasses (https://singlecell.broadinstitute.org/single_cell/study/SCP509/mouse-retinal-ganglion-cell-adult-atlas-and-optic-nerve-crush-time-series?genes=Agtr2&cluster=Ctrl_RGCs_ONC_Dataset&spatialGroups=--&annotation=Cluster--group--cluster&subsample=all&tab=scatter#study-visualize. Accessed on 6 June 2023). Thus, Ang-(1-7) also may be exerting effects directly on rNAION-stressed RGCs.

Our study showed overall improvement in visual function as assessed by OptoMotry as well as fVEP amplitudes in Ang-(1-7)-treated rats compared with vehicle-treated rats across all edema levels. As occurs in patients with spontaneous NAION, rNAION-affected animals exhibit varying levels of ONH edema, with the animals with the greatest severity in ONH edema having poorer visual function [45]. Analysis of the primate NAION (pNAION) model also has revealed a close correlation between the severity of ONH edema and the severity of visual function loss as assessed by fVEP amplitudes [45]. In the rNAION model, the severity of ONH edema correlates with the severity of the rNAION lesion (see Figure 2A in [23]). We used these differences in rNAION-induced ONH edema to segregate animals into mild (450–599 µm), moderate (600–699 µm) and severe (700–850 µm) groups. Ang-(1-7) exhibited its greatest relative neuroregenerative/neuroreparative effects in animals with the most severe ONH edema (see Figure 3 and Figure 4), with lesser relative improvement effects in animals with mild edema.

The level of visual improvement is not explained entirely by single differences in either RGC survival (Ang-(1-7) treatment improved RGC survival by a nonsignificant 10% compared with the vehicle) or a general increase in post-rNAION ON inflammatory cell numbers, as measured by Iba-1 (~2%). Ang-(1-7)/Mas1 activity has been associated with a variety of neuroprotective/neuroregenerative features, including a reduction in astrocyte-related inflammation [46], and a reduction in microglial pro-inflammatory cytokines [47]. AngII activity has also been implicated in the maintenance of the blood–brain barrier [48] This suggests that improved visual function associated with Ang-(1-7) treatment may be due to either a combination of factors or unmeasured factors, such as enhanced function in residual (post-rNAION) neurons, changes in ONH scarring, alteration in both astrocytic and macrophage-related inflammatory responses (M1-neurodegenerative to M2-neuroprotective, or changes in other cell responses resulting in increased ON efficiency. Additionally, while it is possible that Ang-(1-7) treatment could also postpone RGC death after axonal ischemia further than 1 month, this does not explain the robust functional (fVEP) improvement in Ang-(1-7)-treated animals at 28-30d, compared with the nonsignificant RGC loss. A previous study revealed that RGC death in the rNAION model has two peaks: at 10–11 and 20 days, with a return to baseline at 30 days [49]. It is unlikely that additional slow RGC death would explain the differences in function.

Treatment with Ang-(1-7) and other Ang1-7 type agonists represents a new approach to effective clinical treatment of NAION and other forms of white matter stroke. The direct translation of this agent into the clinics is also supported by Ang-(1-7)’s already proven margin of safety in various clinical trials. Further work will focus on identifying the effective treatment time window, clinical effectiveness, and mechanisms associated with functional improvement.

## 5. Conclusions

Ang-(1-7) is the first agent that can be demonstrated to be effective in the rNAION/isolated white matter stroke model when administered systemically at least one day after stroke induction and continued for 21 days. This effect is confirmed by both visual acuity and optic nerve function. While much work remains to be completed concerning overall mechanisms of action and effective treatment time window, its relative effectiveness is more pronounced the greater the degree of lesion severity. Given the low toxicity found in previous clinical studies, Ang-(1-7) holds promise to be a clinically relevant approach to NAION treatment.

## Data Availability

The original contributions presented in this study are included in the article. Further inquiries can be directed to the corresponding author.
